# Intermediate Hyperglycemia Increases the Risk of All-Cause Mortality in Premature Coronary Artery Disease Patients Undergoing Percutaneous Coronary Intervention

**DOI:** 10.31083/j.rcm2412352

**Published:** 2023-12-13

**Authors:** Ziyou Zhou, Linfang Qiao, Yihang Ling, Yibo He, Tian Chang, Hongyu Lu, Sijia Yu, Jin Liu, Wei Guo, Shiqun Chen, Yong Liu, Jiyan Chen

**Affiliations:** ^1^School of Medicine, South China University of Technology, 510006 Guangzhou, Guangdong, China; ^2^Department of Cardiology, Guangdong Provincial People's Hospital (Guangdong Academy of Medical Sciences), Southern Medical University, 510080 Guangzhou, Guangdong, China; ^3^Guangdong Provincial Key Laboratory of Coronary Heart Disease Prevention, Guangdong Cardiovascular Institute, Guangdong Provincial People’s Hospital, Guangdong Academy of Medical Sciences, 510080 Guangzhou, Guangdong, China; ^4^The Second School of Clinical Medicine, Southern Medical University, 510515 Guangzhou, Guangdong, China; ^5^Guangdong Provincial Geriatrics Institute, Guangdong Provincial People’s Hospital, Guangdong Academy of Medical Sciences, 510080 Guangzhou, Guangdong, China; ^6^Global Health Research Center, Guangdong Provincial People’s Hospital, Guangdong Academy of Medical Science, 510100 Guangzhou, Guangdong, China

**Keywords:** premature coronary artery disease, intermediate hyperglycemia, mortality, percutaneous coronary intervention

## Abstract

**Background:**

Hyperglycemia has been associated with an adverse prognosis in patients with premature coronary artery disease (CAD). However, whether the intermediate hyperglycemia status affects the risk of mortality in premature CAD patients treated with percutaneous coronary intervention (PCI), remains unclear.

**Methods:**

We retrospectively included 14,585 premature CAD patients undergoing PCI from 2007 to 2020. Patients were divided into normal glycemia (<6%), intermediate hyperglycemia (6%–6.5%), and hyperglycemia (≥6.5%) according to hemoglobin A1c (HbA1c) level in whole blood. Follow-up all-cause mortality was defined as a primary outcome, and Cox proportional regression analysis was used to assess the association between glycemia status and the primary outcome.

**Results:**

Among 14,585 premature CAD patients undergoing PCI (mean age 43.6 ± 7.6 years, 28.1% female), 2856 (19.6%) were diagnosed with intermediate hyperglycemia. Over a median follow-up of 4.62 years (2.72–7.19 years), patients with hyperglycemia were correlated with higher risk (hazard ratio [HR] 1.35, 95% confidence interval [CI] 1.19–1.54, *p* < 0.001) while patients with intermediate hyperglycemia were associated with intermediate mortality risk from all causes (HR 1.17, 95% CI 1.0–1.36, *p* = 0.049).

**Conclusions:**

Intermediate hyperglycemia was positively associated with all-cause mortality risk in patients with premature CAD undergoing PCI. Active glucose-lowering therapy may be considered in these patients.

**Clinical Trial Registration:**

NCT05050877.

## 1. Introduction

Coronary artery disease (CAD) represents one of the principal causes of death and morbidity globally and the most important cause of premature death in the developed world [1]. Young CAD patients with symptoms (i.e., premature CAD) accepting percutaneous coronary intervention (PCI), were more likely to have acute myocardial infarction, unstable angina, out-of-hospital cardiac arrest, and cardiac shock compared with elderly patients [2]. The long-term morbidity or mortality of premature CAD patients has not improved over the previous decades, accompanied by an increasing burden of cardiovascular risk factors [3]. In addition, current studies revealed that premature CAD is often accompanied by ischemic recurrence and has a high proportion of cardiovascular risk factors, which were modifiable but frequently failed to control [4]. The control of risk factors is therefore particularly important in premature CAD patients to improve their prognosis.

It was reported that 36% of premature CAD patients develop recurrent major adverse cardiovascular events (MACE) at least twice, while diabetes is a major exposure factor for the recurrence of MACE [5]. 2019 European Society of Cardiology (ESC) Guidelines highlighted the prevention and control of risk factors as of great significance for people suffering CAD, especially for patients accompanied with diabetes requiring active control of hemoglobin A1c (HbA1c) [6]. However, the target of glycemic control for premature CAD patients remains undetermined. On the other hand, previous studies have shown that intensive glycemic control to keep HbA1c below 6.5% can reduce macrovascular and microvascular events by 10% [7]. Patients with intermediate hyperglycemia of HbA1c ranging from 6.0 to 6.5% were defined as prediabetic according to the World Health Organization (WHO) [8]. Compared with normoglycemic individuals, prediabetes is related to a high atherosclerotic burden and the number of vessels affected in Ahmed’s study [9]. Another study illustrated that patients with prediabetes have a significantly increased long-term risk of cardiovascular disease after PCI [10]. However, Juan Francisco Cueva-Recalde *et al*. [11] reported that prediabetes shows no association with higher mortality or adverse cardiovascular outcomes in CAD patients undergoing PCI. Whether the intermediate hyperglycemia of HbA1c range from 6.0–6.5% should be treated in premature CAD patients with or without diabetes is unclear yet.

Given the paucity of data on the association between the glycemic control target and prognosis among patients, the study aims to assess the prevalence of intermediate hyperglycemia and its effect on long-term all-cause death in premature CAD patients treated with PCI.

## 2. Materials and Methods

### 2.1 Study Population

**Fig. 1.**
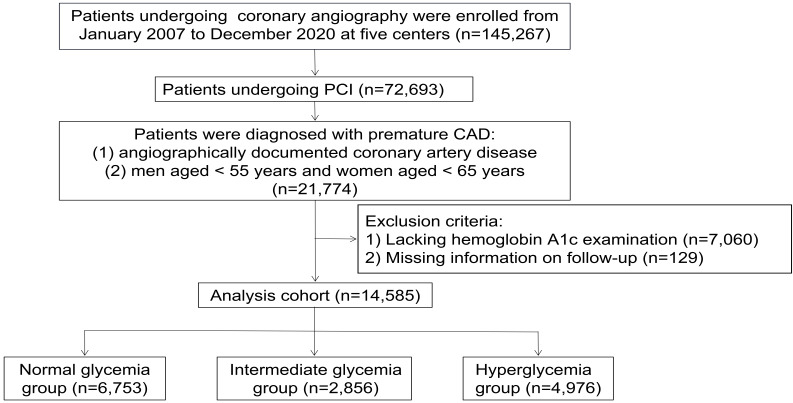
**The flow of participants through the trial**. PCI, percutaneous coronary intervention; CAD, coronary artery disease.

The research conducted was a retrospective, observational investigation utilizing data from the registry of Cardiorenal ImprovemeNt II (CIN-II, NCT05050877) cohort, which enrolled consecutive patients undergoing coronary angiography at five tertiary teaching hospitals in southern China from January 2007 to December 2020. A total of 21,774 patients diagnosed with premature CAD undergoing PCI were included, in which patients diagnosed with stenosis ≥50% in at least one of the coronary arteries documented by coronary angiography in men aged <55 years old and women aged <65 years old, were defined as premature CAD. After excluding patients without baseline HbA1c and missing information on follow-up outcomes, 14,585 patients were eventually included in the study (Fig. 1). The study adhered to the principles outlined in the “Declaration of Helsinki” and received ethical approval from the Ethics Research Committee of Guangdong Provincial People’s Hospital (Approval No. GDREC2012141H).

### 2.2 Data Collection and Definition

The data for the study were sourced from the Electronic Clinical Management System utilized by the hospitals involved in the research. The baseline information mainly included demographic characteristics, medical history, medications at discharge, laboratory examination and other clinical characteristics.

Per the criteria established by the International Diabetes Expert Committee (in accordance with the World Health Organization guidelines), glycemic status was categorized as follows: [8]. (i) normal HbA1c, less than 6.0%; (ii) prediabetes, ranging from 6.0% to 6.4%; and (iii) diabetes, equal to or greater than 6.5%. Based on the baseline HbA1c levels in whole blood instead of disease status, the patients were classified into three distinct groups: (i) normal glycemia group [HbA1c level was <6.0%], (ii) intermediate hyperglycemia [HbA1c level ranging from 6.0 to 6.4%] and (iii) hyperglycemia group [HbA1c level was ≥6.5%]. Diabetes mellitus (DM) was defined as self-reported history of type 1 or type 2 diabetes, use of any glucose-lowering medication or documented HbA1c value of ≥6.5%. Three glycemic status groups defined based on fasting blood glucose (FBG) can be found in the supplementary document. Chronic kidney disease (CKD) was defined as an estimated glomerular filtration rate (eGFR) of less than 60 mL/min per 1.73 $m^{2}$ present [12]. Hypertension, acute myocardial infarction (AMI) and atrial fibrillation (AF) were defined according to the 10th Revision Codes of the International Classification of Diseases (ICD-10). Congestive heart failure (CHF) was defined as New York Heart Association class >2 or Killip class >1 according to documented records or discharge diagnosis by clinical physicians. Anemia was defined as a hematocrit value of <39% for men or <36% for women following the World Health Organization criteria.

### 2.3 Clinical Outcomes

The primary endpoint was long-term all-cause mortality. The secondary endpoint was cardiac mortality, defined as any death due to any cardiac cause. The follow-up information was acquired by matching the survival data from the Centers for Disease Control and Prevention.

### 2.4 Statistical Analysis

Continuous variables were presented as mean ± standard deviation if they followed a normal distribution or as median with interquartile range if non-normally distributed. Categorical variables were presented as counts and percentages. Group comparisons were conducted using either the Student *t*-test or the Wilcoxon Rank Sum test for continuous variables, and the chi-square test or Fisher exact test for categorical variables, as appropriate.

The association between glycemia status and long-term all-cause mortality as well as cardiac mortality was evaluated by the Cox proportional-hazards model, presented as hazard ratio (HR) and 95% confidence interval (CI). Adjusted covariates included age, sex, CKD, hypertension, CHF, AF, AMI, cholesterol, and low-density lipoprotein cholesterol (LDL-C) according to the significance in the univariate analysis, clinical practice and previous studies. The time-to-endpoint data were presented graphically using Kaplan-Meier (K-M) curves, and we used restricted cubic splines (RCS) to explore the linear relationship between HbA1C and hazard ratios (HRs) for all-cause mortality in premature CAD with PCI.

## 3. Results

### 3.1 Baseline Characteristics

Among the 14,585 premature CAD patients undergoing PCI, the majority were male (71.9%), and the mean age was 50.4 ± 7.2 years. Generally, 5047 (34.7%) patients were complicated with AMI, and 4598 (36.3%) patients had multi-vessel lesions. 2044 (14.1%) patients with congestive heart failure, 6928 (47.6%) patients with hypertension, and 1317 (9%) patients with CKD. For patients categorized according to HbA1c value, 6753 (46.3%) patients were normal glycemia (average HbA1c: 5.49), 2856 (19.6%) patients were intermediate hyperglycemia (average HbA1c: 6.17) and 4976 (34.1%) were hyperglycemia (average HbA1c: 8.34). When comparing with the normal glycemia group, patients in the intermediate hyperglycemia and hyperglycemia groups exhibited tendencies towards being older, female, and having higher levels of triglycerides and cholesterol. Patients in the intermediate hyperglycemia and hyperglycemia groups showed a higher incidence of comorbidities (i.e., hypertension and chronic kidney diseases). Patients with hyperglycemia were more prone to congestive heart failure and atrial fibrillation as well as having a higher rate of taking a prescription of medicine, including angiotensin II receptor blockers (ARBs), beta-blockers, than both the normal glycemia and intermediate hyperglycemia group. More data on baseline characteristics of the study population are shown in Table 1.

**Table 1.** **Baseline characteristics of the study population**.

	Overall	Normal glycemia	Intermediate hyperglycemia	Hyperglycemia	*p*-value
No. of participants	14,585	6753	2856	4976	
Age, years	50.40 (±7.15)	49.07 (±7.21)	51.11 (±6.77)	51.78 (±6.97)	<0.001
Female, %	4101 (28.1)	1468 (21.7)	855 (29.9)	1778 (35.7)	<0.001
Hypertension, %	6928 (47.6)	2799 (41.6)	1408 (49.5)	2721 (54.8)	<0.001
Diabetes mellitus, %	4577 (34.1)	332 (4.9)	429 (15.0)	3816 (100.0)	<0.001
Congestive heart failure, %	2044 (14.1)	843 (12.5)	376 (13.2)	825 (16.6)	<0.001
Atrial fibrillation, %	202 (1.4)	71 (1.1)	44 (1.5)	87 (1.8)	0.005
Acute myocardial infraction, %	5047 (34.7)	2482 (36.9)	882 (31.0)	1683 (33.9)	<0.001
Multi-vessel disease, %	4598 (36.3)	1862 (32.4)	950 (36.9)	1786 (41.3)	<0.001
2-vessel disease, (%)	993 (6.9)	468 (7.0)	187 (6.6)	338 (7.0)	0.826
3-vessel disease, (%)	3605 (28.5)	1394 (24.2)	763 (29.6)	1448 (33.5)	<0.001
Chronic kidney disease, %	1317 (9.0)	448 (6.6)	244 (8.5)	625 (12.6)	<0.001
Stroke, %	88 (0.6)	32 (0.5)	28 (1.0)	28 (0.6)	0.012
Anemia, %	2946 (20.5)	1202 (18.1)	569 (20.2)	1175 (24.0)	<0.001
FBG, mmol/L	5.99 (±2.28)	4.91 (±0.59)	6.50 (±0.26)	10.17 (±2.82)	<0.001
Album, g/L	38.37 (±4.30)	38.70 (±4.13)	38.43 (±4.10)	37.89 (±4.59)	<0.001
Triglyceride, mmol/L	2.03 (±1.64)	1.81 (±1.34)	1.96 (±1.28)	2.36 (±2.09)	<0.001
Cholesterol, mmol/L	4.83 (±1.37)	4.78 (±1.36)	4.87 (±1.34)	4.87 (±1.40)	0.001
HDL-C, mmol/L	0.98 (±0.26)	1.00 (±0.26)	0.99 (±0.25)	0.96 (±0.25)	<0.001
LDL-C, mmol/L	3.07 (±1.10)	3.06 (±1.10)	3.11 (±1.12)	3.05 (±1.08)	0.056
Hemoglobin A1c, %	6.59 (±1.67)	5.49 (±0.38)	6.17 (±0.14)	8.34 (±1.77)	<0.001
eGFR, mL/min/1.73 $m^{2}$	88.85 [75.45, 104.37]	89.10 [76.87, 103.34]	88.01 [75.55, 102.42]	89.15 [72.94, 107.06]	0.095
Uric acid, mmol/L	389.79 (±108.48)	391.05 (±105.87)	396.98 (±103.60)	383.95 (±114.29)	<0.001
Neutrophil counts, ${\mathrm{10}}^{9}$/L	5.72 (±3.01)	5.77 (±3.10)	5.46 (±2.84)	5.80 (±2.98)	<0.001
Lymphocyte counts, ${\mathrm{10}}^{9}$/L	2.09 (±0.75)	2.03 (±0.73)	2.16 (±0.75)	2.14 (±0.76)	<0.001
Statins, %	13705 (96.2)	6351 (96.3)	2737 (97.0)	4617 (95.6)	0.005
ACEI, %	6952 (48.8)	3303 (50.1)	1412 (50.1)	2237 (46.3)	<0.001
ARB, %	3392 (23.8)	1366 (20.7)	658 (23.3)	1368 (28.3)	<0.001
Aspirin, %	13943 (97.9)	6458 (98.0)	2758 (97.8)	4727 (97.9)	0.815
β-blocker, %	12299 (86.4)	5621 (85.3)	2458 (87.1)	4220 (87.4)	0.002
Calcium channel blocker, %	2965 (20.8)	1350 (20.5)	550 (19.5)	1065 (22.0)	0.019
Diuretics, %	1335 (9.4)	496 (7.5)	211 (7.5)	628 (13.0)	<0.001
Oral antidiabetic drug, %	2974 (20.9)	133 (2.0)	266 (9.4)	2575 (53.3)	<0.001
Insulin, %	670 (4.7)	99 (1.5)	36 (1.3)	535 (11.1)	<0.001

Values are mean ± SD, n (%) and median (interquartile range). Abbreviation: FBG, fasting blood glucose; HDL-C, high-density lipoprotein cholesterol; LDL-C, low-density lipoprotein cholesterol; eGFR, estimated glomerular filtration rate; ACEI, angiotensin-converting enzyme inhibitors; ARB, angiotensin II receptor blocker; SD, standard deviation.

### 3.2 Long-Term Outcome 

**Fig. 2.**
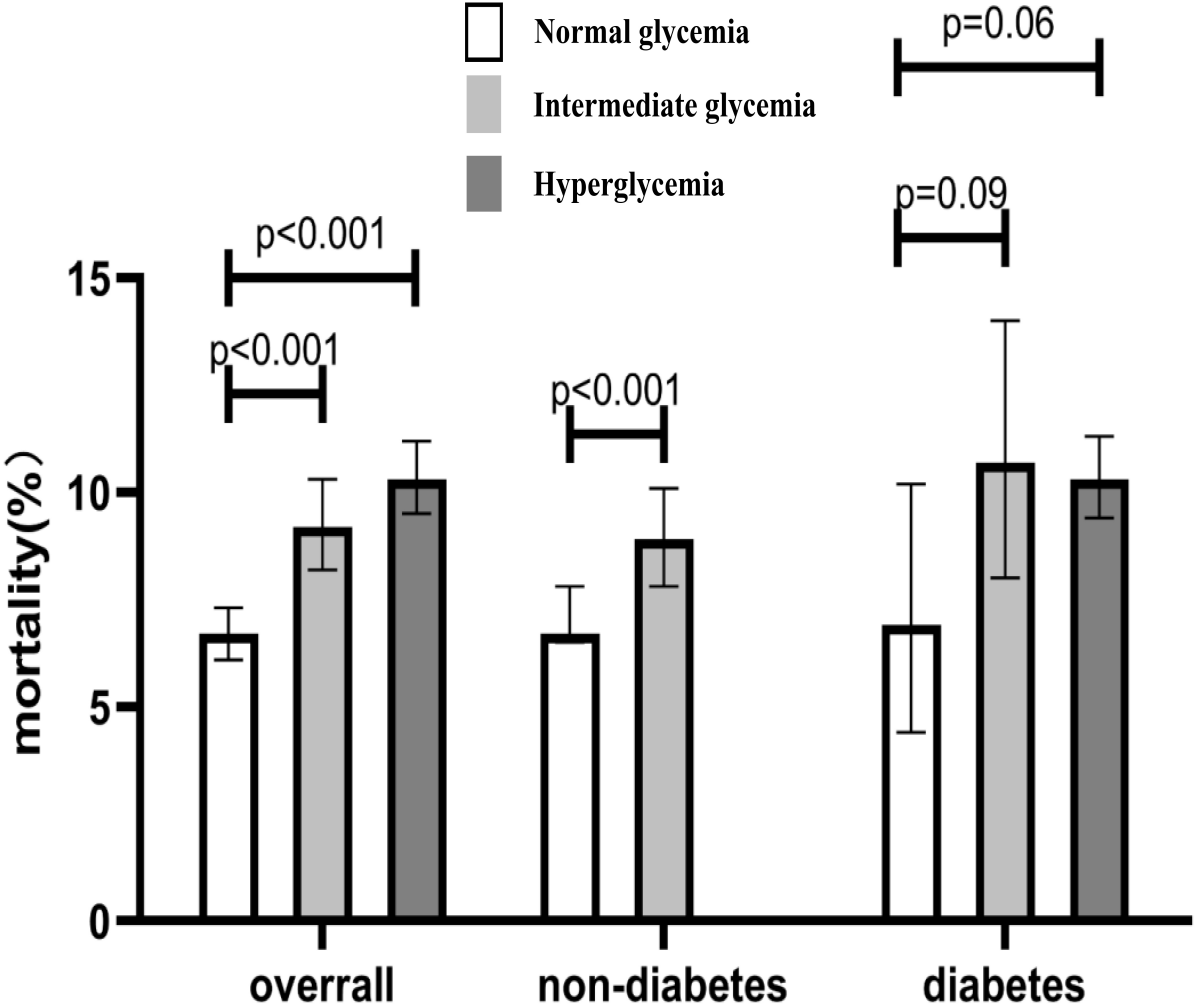
**All-cause death rates for patients with different glycemic status**.

**Fig. 3.**
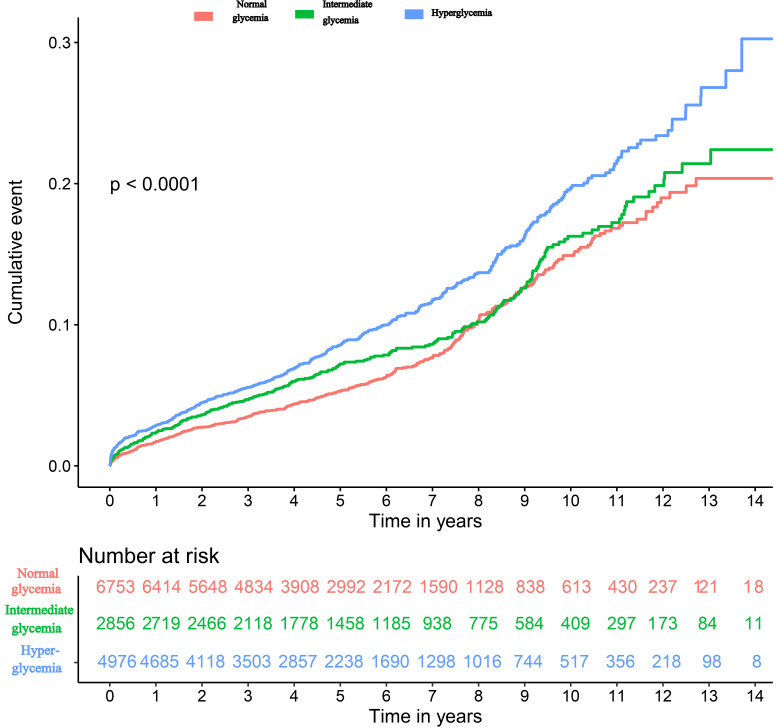
**Kaplan-Meier curves of long-term all-cause mortality**.

Over a median follow-up of 4.62 years (interquartile range 2.72–7.19 years), 1231 (8.44%) patients died. The all-cause mortality of overall patients in the intermediate hyperglycemia group and hyperglycemia group were significantly higher than in the normal glycemia group (*p* < 0.001). When categorized based on the presence of diabetes, the rate of all-cause death was found to be higher in non-diabetic patients with intermediate hyperglycemia than in patients with normal glycemia status. However, there was no statistical significance of all-cause mortality between persons with diabetes with intermediate hyperglycemia status and those with normal glycemia status (*p* = 0.09). Persons with diabetes with hyperglycemia appeared to have higher all-cause mortality than those with normal glycemia status, of borderline statistical significance (*p* = 0.06) (Fig. 2). Kaplan-Meier curves for cumulative survival based on the tertiles of baseline HbA1c were presented in Fig. 3.

**Fig. 4.**
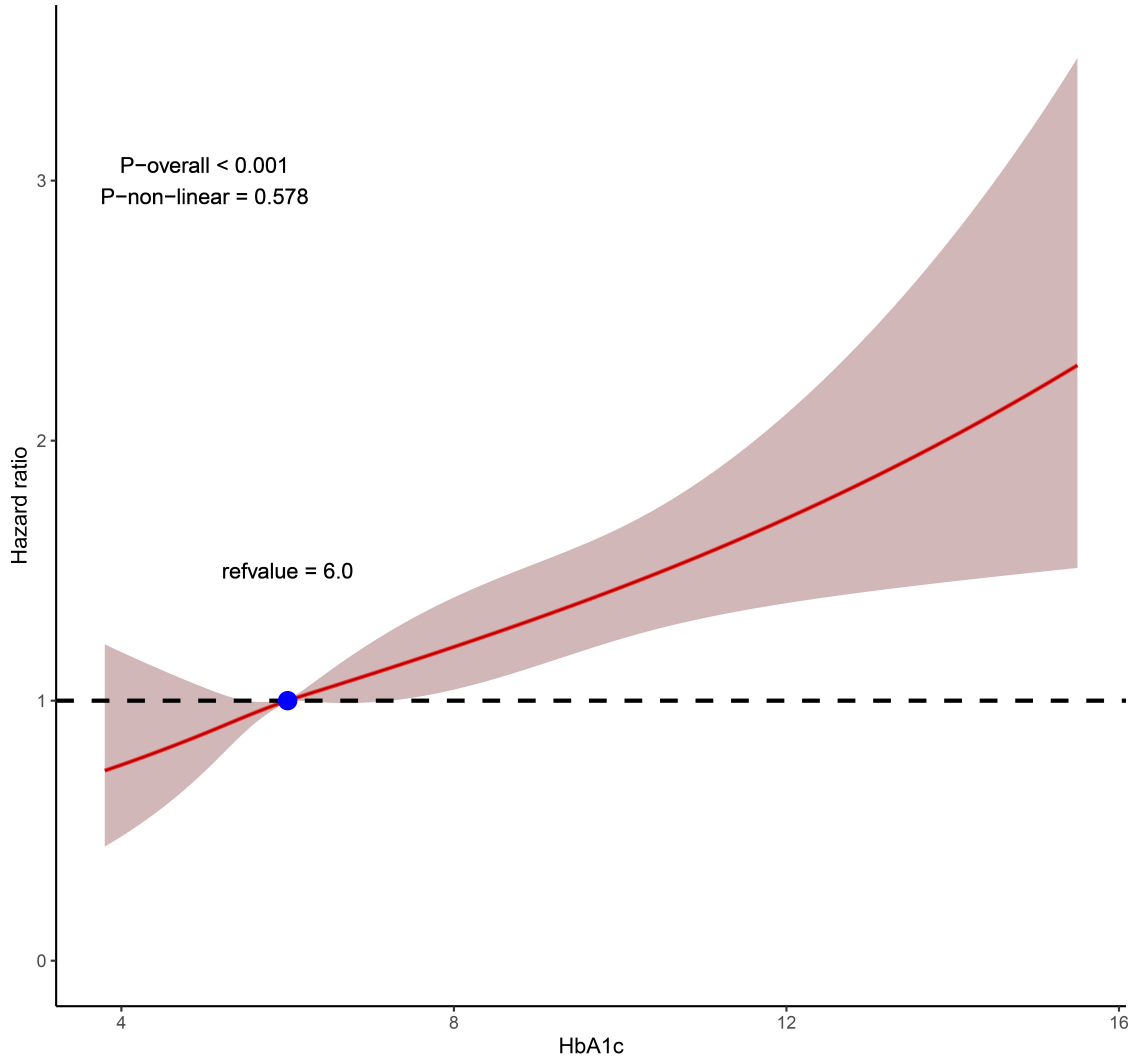
**Restricted cubic splines for the relationship between HbA1c and all-cause mortality**. HbA1c, hemoglobin A1c.

When adjusted for confounders, the Cox regression model indicated that premature CAD patients undergoing PCI with intermediate hyperglycemia were related to a 17% elevated risk of all-cause mortality, while hyperglycemia was associated with a 35% increased risk of all-cause mortality (adjusted hazard ratio for intermediate hyperglycemia group and hyperglycemia group, respectively: 1.169 [95% confidence interval (CI): 1.001–1.364], *p* = 0.049; and 1.352 [95% CI: 1.185–1.543], *p* < 0.001) (Table 2). When using cardiovascular death as the endpoint, the hyperglycemia group still demonstrated an increased risk of cardiovascular mortality (**eTable 1** in the **Supplementary Material**), while the hyperglycemia group (defined by fasting blood glucose) had a similar association with the all-cause mortality (**eTable 2** in the **Supplementary Material**). Restricted cubic spline curves demonstrated that the elevation of HbA1c value was collinearly linked to the heightened risk of all-cause death among premature CAD patients undergoing PCI (*p* < 0.001; *p* for non-linearity = 0.578) (Fig. 4).

**Table 2.** **Hazard ratios of all-cause mortality by measures of HbA1c**.

	Model 1		Model 2		Model 3	
	HR	*p*-value	HR	*p*-value	HR	*p*-value
Normal glycemia	1 (ref)	-	1 (ref)	-	1 (ref)	-
Intermediate hyperglycemia	1.174 (1.008–1.367)	0.039	1.167 (1.002–1.360)	0.047	1.169 (1.001–1.364)	0.049
Hyperglycemia	1.483 (1.307–1.683)	<0.001	1.478 (1.300–1.680)	<0.001	1.352 (1.185–1.543)	<0.001

Model 1: unadjusted. Model 2: adjusted for age, gender. Model 3: adjusted for age, gender, cholesterol, low-density lipoprotein cholesterol, acute myocardial infraction, congestive heart failure, atrial fibrillation, hypertension, chronic kidney disease. HbA1c, hemoglobin A1c; HR, hazard ratio.

## 4. Discussion

Overall, in this multi-center retrospective study, we observed that one-third of patients had hyperglycemia, and intermediate hyperglycemia was present in 19.6% of our cohort. According to our results, we found that among premature CAD patients treated with PCI, intermediate hyperglycemia status is associated with a 17% increased risk of all-cause mortality. The finding reinforces the importance of strictly controlling HbA1c among individuals who present with CAD at a young age.

Premature CAD represents a large proportion of overall cardiovascular disease, and the rate of mortality has stalled despite an overall declining global cardiovascular mortality rate, probably due to inadequate control of risk factors [3, 13, 14]. Our study discovered a high prevalence of hypertension and DM in the study population and the mean levels of LDL-C were much higher than the guideline-recommended goal [15, 16]. The previous study confirmed that premature CAD, as a majority cardiovascular disease in the young, was significantly and positively associated with some risk factors, including DM, family history of CAD, dyslipidemia, smoking, and hypertension, which could lead to poor prognoses [5, 17]. In addition to hyperglycemia, our study demonstrated that intermediate hyperglycemia was associated with increased mortality in premature CAD patients undergoing PCI as well. Therefore, in parallel to the traditional risk factors like smoking and dyslipidemia, intermediate hyperglycemia may need to be controlled in these young patients imminently.

HbA1c has been suggested as a dependable tool not only for diagnosing DM but also for assessing the risk of cardiovascular events in both DM and non-DM patients [18, 19]. A previous study concluded that HbA1c exhibited a stronger association with the risks of cardiovascular disease and all-cause mortality when compared to fasting glucose [20]. In Adam’s study, compared with the oral glucose tolerance test (OGTT), HbA1c performed better for the predictive power of cardiovascular disease and chronic kidney disease [21]. Some studies demonstrated that OGTT is more sensitive than HbA1c in detecting hyperglycemia in CAD patients [22, 23]. However, the results of OGTT could be influenced by some factors such as the pre-test carbohydrate diet, physical activity, as well as the severity of the myocardial injury [24]. The 2019 European Society of Cardiology guidelines recommend that screening for diabetes in patients with cardiovascular disease should start with glycosylated hemoglobin [25]. Our study also identified a nearly linear relationship between HbA1c levels and all-cause mortality, suggesting that using HbA1c alone is a viable approach to assess the glycemic status of these patients. Benefiting from the stability of HbA1c and the convenience due to no testing time limitations, routine screening for HbA1c in young patients with CAD may be necessary.

This long-term follow-up study of premature CAD patients undergoing PCI showed that half of the patients’ HbA1c was over 6%, and the HbA1c level of one-third of patients was even more than 6.5%. The 2019 ESC Guidelines suggested monitoring HbA1c in chronic coronary syndrome patients and keeping it below 6.5% [6, 7]. However, as more attention is drawn to prediabetes, studies have found that the risk of cardiovascular events is elevated in the group without diabetes with HbA1c, 5.7–6.4% [26, 27]. In the group without diabetes with HbA1c of 6.0–6.5% (i.e., prediabetes), we found that the all-cause mortality of the group was higher than those with normal glycemia, which is consistent with several meta-analyses [27, 28]. Hence, our study also confirmed the harm of intermediate hyperglycemia in the group without diabetes.

Interestingly, compared with well-controlled persons with diabetes (HbA1c <6%), the all-cause mortality of persons with diabetes with intermediate hyperglycemia and hyperglycemia were higher in our study, but the difference did not reach statistical significance. Similar results to our study was seen in another study of the CAD population [19]. One possible reason for this result is that patients suffering from DM are more likely to receive lifestyle modification counselling and secondary prevention, especially poorly controlled patients whose HbA1c remains greater than 6.5% after treatment. In addition, they are prone to receiving guideline-recommended cardiovascular medication, which could lower the risk of adverse events. However, considering the absence of data on glycemic control, whether the premature CAD patients treated with PCI that we included had proper anti-diabetic therapy remains unclear, which warrants further study. Furthermore, it was confirmed that persons with HbA1c levels below 5% had the lowest mortality rates, and each 1-percentage point increase in HbA1c was associated with a relative risk for all-cause mortality [29]. Therefore, we believed that patients were at an enhanced risk of death in this possibly overlooked intermediate hyperglycemia interval. This suggests that the control target of HbA1c in premature CAD patients undergoing PCI should be more restrictive, regardless of the presence of diabetes.

Despite the accumulating evidence suggesting an association between elevated levels of HbA1c and DM with a poor outcome in premature AMI patients [30, 31, 32], the target of HbA1c in premature CAD patients undergoing PCI has not been well established. Alfredo Caturano *et al*. [33] reviewed the potential mechanism of strict glycemic control in cardiovascular protection in patients with acute coronary syndrome, mainly including anti-apoptotic actions, anti-inflammatory effects, increasing nitric oxide, reducing plasma level of free fat acid, etc. Considering hypoglycemia, past clinical trials have published contradictory conclusions on whether patients with acute coronary syndrome should strictly control glycemia. However, with the application of Glucagon-like peptide-1 receptor agonists and sodium-glucose cotransporter-2 inhibitors, patients may not need to bear the risk of hypoglycemia and could benefit from their cardiovascular protection [33].

Intermediate hyperglycemia defined by FBG increased all-cause mortality but did not reach statistical significance in supplementary analyses. Still, there are some rationalities to the result. First, the FBG samples should be obtained after at least 8 hours of fasting, which is a considerable practical problem, leading to a large missing rate in our study. Of the 14,585 premature CAD patients undergoing PCI we included, 5571 (38.2%) patients had missing FBG information. Second, FBG may be affected by many factors, including unstable sample, recent exercise, and acute stress. Patients we included were more likely to suffer from acute stress [34]. Third, FBG is less tightly linked to diabetes complications than HbA1C, while CAD is one of the major diabetes complications [35]. Thus, though HbA1C may be affected by anemia, pregnancy, hypertriglyceridemia, and chronic liver disease, it’s still worth using HbA1C to be a prognostic index considering it has very little biological variability.

During 4.62 years of median follow-up, our study showed that intermediate hyperglycemia status, defined as HbA1c 6.0–6.5%, still increases the risk of all-cause mortality in the large cohort of premature CAD patients undergoing PCI with or without DM. Therefore, active lifestyle modification and aggressive treatment should be considered for patients with premature CAD and glycemic disturbances.

### Limitation

Firstly, our results were subject to the limitation of the retrospective design. However, this allowed us to examine a large number of individuals with CAD at a young age in several Chinese regional central tertiary teaching hospitals. Further prospective studies focusing on premature CAD patients are needed. Secondly, despite having controlled for some common confounding factors, there may still be some potential confounding factors, such as the number of vessels treated by PCI that haven’t been fully adjusted for. Thirdly, we chose to focus solely on all-cause mortality as the primary endpoint due to limited data, and therefore, did not include other endpoints such as major adverse cardiovascular events, heart failure, failure of target vessel revascularization, or cardiovascular re-hospitalization in our study. However, all-cause mortality is still worth being the primary endpoint for its accuracy and accessibility. More national studies are necessary to better understand the link between hyperglycemia status and adverse cardiovascular outcomes. Finally, we did not observe the variability of HbA1c in follow-up [36], and identified hyperglycemia based solely on baseline HbA1c, but it is more applicable to routine screening for timely detection to draw the attention of clinical doctors.

## 5. Conclusions

In conclusion, we confirmed that compared with normal glycemia, patients with intermediate hyperglycemia are associated with a 17% residual risk of all-cause mortality, while hyperglycemia is associated with a 35% increased risk in premature CAD patients undergoing PCI. Our findings highlight the need for monitoring HbA1c earlier in patients with premature CAD to prevent future adverse prognoses. Active hypoglycemic therapy and lower HbA1c target should be considered in these patients.

## Data Availability

The datasets used and/or analyzed during the current study are available from the corresponding author on reasonable request.

## Abbreviations

CAD, coronary artery disease; PCI, percutaneous coronary intervention; MACE, major adverse cardiovascular events; HbA1C, hemoglobin A1c; CKD, chronic kidney disease; eGFR, estimated glomerular filtration rate; AMI, acute myocardial infarction; HDL-C, high-density lipoprotein cholesterol; LDL-C, low-density lipoprotein cholesterol; ACEI, angiotensin-converting enzyme inhibitors; ARB, angiotensin II receptor blocker.

## Supplementary Material

Supplementary material associated with this article can be found, in the online version, at https://doi.org/10.31083/j.rcm2412352.
