# Supplementary material for: Intermediate Hyperglycemia Increases the Risk of All-Cause Mortality in Premature Coronary Artery Disease Patients Undergoing Percutaneous Coronary Intervention
Source: Rev Cardiovasc Med. 2023 Dec 13;24(12):352. doi: 10.31083/j.rcm2412352 (PMC11272885; doi:10.31083/j.rcm2412352)
Supplement: Supplementary file 1 [file 2153-8174-24-12-352-s1.zip › 2153-8174-24-12-352-s1.docx]

**eTable 1. Hazard ratios of cardiac mortality by measures of HbA1c**

|  | **Events, No. (%)** | **Model 1** | | **Model 2** | | | **Model 3** | | |
| --- | --- | --- | --- | --- | --- | --- | --- | --- | --- |
|  |  | HR | P-value | | HR | P-value | | HR | P-value |
| Normal glycemia | 131(1.9) | 1 (ref) | - | | 1 (ref) | - | | 1 (ref) | - |
| Intermediate hyperglycemia | 80(2.8) | 1.24 (0.939-1.639) | 0.13 | | 1.228 (0.929-1.625) | 0.149 | | 1.213 (0.911-1.614) | 0.187 |
| Hyperglycemia | 246(4.9) | 2.466 (1.995-3.049) | <0.001 | | 2.433 (1.961-3.018) | <0.001 | | 1.352 (1.185-1.543) | <0.001 |

Model 1: unadjusted

Model 2: adjusted for age, gender

Model 3: adjusted for age, gender, cholesterol, low-density lipoprotein cholesterol, chronic kidney disease, congestive heart failure, hypertension, atrial fibrillation, acute myocardial infraction

(i) normal glycemia group [HbA1c level was < 6.0%], (ii) intermediate hyperglycemia [HbA1c level ranging from 6.0 to 6.4%] and (iii) hyperglycemia group [HbA1c level was ≥ 6.5%]

**eTable 2. Hazard ratios of all-cause mortality by measures of fasting blood glucose**

|  | **Model 1** | | **Model 2** | | **Model 3** | |
| --- | --- | --- | --- | --- | --- | --- |
|  | HR | P-value | HR | P-value | HR | P-value |
| Normal glycemia | 1 (ref) | - | 1 (ref) | - | 1 (ref) | - |
| Intermediate hyperglycemia | 1.24(0.939-1.639) | 0.13 | 1.228(0.929-1.625) | 0.149 | 1.213(0.911-1.614) | 0.187 |
| Hyperglycemia | 2.466(1.995-3.049) | <0.001 | 2.433(1.961-3.018) | <0.001 | 1.352(1.185-1.543) | <0.001 |

Model 1: unadjusted

Model 2: adjusted for age, gender

Model 3: adjusted for age, gender, cholesterol, low-density lipoprotein cholesterol, chronic kidney disease, congestive heart failure, hypertension, atrial fibrillation, acute myocardial infraction

According to the World Health Organization criteria, glycemic status was defined as: (i) normal fasting glucose, FBG ≤ 6.0 mmol/L; (ii) impaired fasting glucose, 6.1 ≤ FBG ≤ 6.9mmol/L; and (iii) diabetes, FBG ≥ 7.0 mmol/L. Patients were also categorized into three groups based on their baseline FBG: (i) normal glycemia group [FBG ≤ 6.0 mmol/L], (ii) intermediate hyperglycemia [6.1 ≤ FBG ≤ 6.9mmol/L] and (iii) hyperglycemia group [FBG ≥ 7.0 mmol/L
